# Electrocardiogram-synchronized pulsatile extracorporeal life support preserves left ventricular function and coronary flow in a porcine model of cardiogenic shock

**DOI:** 10.1371/journal.pone.0196321

**Published:** 2018-04-24

**Authors:** Petr Ostadal, Mikulas Mlcek, Holger Gorhan, Ivo Simundic, Svitlana Strunina, Matej Hrachovina, Andreas Krüger, Dagmar Vondrakova, Marek Janotka, Pavel Hala, Martin Mates, Martin Ostadal, James C. Leiter, Otomar Kittnar, Petr Neuzil

**Affiliations:** 1 Cardiovascular Center, Na Homolce Hospital, Prague, Czech Republic; 2 Department of Physiology, First Faculty of Medicine, Charles University in Prague, Prague, Czech Republic; 3 Xenios AG, Heilbronn, Germany; 4 Faculty of Biomedical Engineering, Czech Technical University in Prague, Prague, Czech Republic; 5 Deparment of Orthopedics, Na Bulovce Hospital, Prague, Czech Republic; 6 Department of Molecular and Integrative Biology, Geisel School of Medicine at Dartmouth, Lebanon, New Hampshire, United States of America; Scuola Superiore Sant’Anna, ITALY

## Abstract

**Introduction:**

Veno-arterial extracorporeal life support (ECLS) is increasingly being used to treat rapidly progressing or severe cardiogenic shock. However, it has been repeatedly shown that increased afterload associated with ECLS significantly diminishes left ventricular (LV) performance. The objective of the present study was to compare LV function and coronary flow during standard continuous-flow ECLS support and electrocardiogram (ECG)-synchronized pulsatile ECLS flow in a porcine model of cardiogenic shock.

**Methods:**

Sixteen female swine (mean body weight 45 kg) underwent ECLS implantation under general anesthesia and artificial ventilation. Subsequently, acute cardiogenic shock, with documented signs of tissue hypoperfusion, was induced by initiating global myocardial hypoxia. Hemodynamic cardiac performance variables and coronary flow were then measured at different rates of continuous or pulsatile ECLS flow (ranging from 1 L/min to 4 L/min) using arterial and venous catheters, a pulmonary artery catheter, an LV pressure-volume loop catheter, and a Doppler coronary guide-wire.

**Results:**

Myocardial hypoxia resulted in declines in mean cardiac output to 1.7±0.7 L/min, systolic blood pressure to 64±22 mmHg, and LV ejection fraction (LVEF) to 22±7%. Synchronized pulsatile flow was associated with a significant reduction in LV end-systolic volume by 6.2 mL (6.7%), an increase in LV stroke volume by 5.0 mL (17.4%), higher LVEF by 4.5% (18.8% relative), cardiac output by 0.37 L/min (17.1%), and mean arterial pressure by 3.0 mmHg (5.5%) when compared with continuous ECLS flow at all ECLS flow rates (P<0.05). At selected ECLS flow rates, pulsatile flow also reduced LV end-diastolic pressure, end-diastolic volume, and systolic pressure. ECG-synchronized pulsatile flow was also associated with significantly increased (7% to 22%) coronary flow at all ECLS flow rates.

**Conclusion:**

ECG-synchronized pulsatile ECLS flow preserved LV function and coronary flow compared with standard continuous-flow ECLS in a porcine model of cardiogenic shock.

## Introduction

Extracorporeal life support (ECLS) in the veno-arterial configuration (VA-ECLS) is an established method that offers circulatory support in the most severe conditions of circulatory failure such as rapidly progressing cardiogenic shock or refractory cardiac arrest [1–3]. Randomized controlled trials confirming the beneficial effect of this therapeutic approach remain lacking despite several ongoing prospective studies [4, 5]. However, favorable outcomes with VA-ECLS therapy have been demonstrated in retrospective studies and case series [6–8].

The basic principle of peripheral VA-ECLS therapy is the insertion of an inflow cannula through the femoral vein into the right atrium and an outflow cannula typically to the femoral artery. After the connection of the cannulas to the ECLS circuit, the venous blood is drained by the extracorporeal blood pump to the oxygenator, where the blood gases are exchanged, and the oxygenated blood is returned to the descending aorta. Although the VA-ECLS configuration offers maintenance of adequate tissue perfusion during circulatory collapse, it may be associated with unfavorable consequences for the failing heart because the outflow component increases left ventricular (LV) afterload [9]. If LV systolic function is already severely compromised, the increased afterload may cause LV overload and further reduction of LV function, even though the right ventricle may be fully unloaded [9–11]. Progressive distension of an overloaded LV is frequently associated with subsequent severe pulmonary edema and results in a critical condition that often requires urgent intervention (eg, implantation of an LV-assist device) [3, 9–12]. The impairment of cardiac performance with increased extracorporeal blood flow (EBF) during ECLS therapy has been well documented in several experimental and clinical studies [13–20].

A novel electrocardiogram (ECG)-synchronized, pulsatile ECLS system has recently been introduced, enabling decreased extracorporeal blood flow during systole and increased flow during diastole, which, ideally should decrease afterload and improve LV function in the setting of ECLS [21, 22]. The aim of our study was to compare the effects of synchronized pulsatile and standard continuous ECLS flow on cardiac performance and coronary blood flow.

## Materials and methods

This study was approved by the Charles University 1^st^ Medical School Institutional Animal Care and Use Committee, and was performed at the Animal Laboratory, Department of Physiology, 1^st^ Medical School, Charles University in Prague and Na Homolce Hospital, Prague, Czech Republic, in accordance with Act No 246/1992 Coll., for the protection of animals against cruelty. The investigation and protocol conformed to the Guide for the Care and Use of Laboratory Animals published by the United States National Institutes of Health (Publication No. 85–23, revised 1985).

### Animal model

Sixteen female swine (*Sus scrofa domestica*, Large White × Landrace crossbreed), four to five months of age and a mean body weight of 45 kg were used. Full details of the animal model used in the present study can be found in previous publications [13, 23].

“Briefly, after a 24 h fast, general anesthesia was induced by administration of midazolam (0.3 mg/kg intramuscular) and ketamine hydrochloride (15 mg/kg to 20 mg/kg intramuscular). Initial propofol and morphine boluses (2 mg/kg intravenous [IV] and 0.1 mg/kg to 0.2 mg/kg IV, respectively) were administered, followed by orotracheal intubation. Continuous IV infusions of propofol (8 mg/kg/h to 10 mg/kg/h) and morphine (0.1 mg/kg/h to 0.2 mg/kg/h) were used to maintain anesthesia. The doses were adjusted according to physiological parameters, pupillary photoreactions, corneal and palpebral reflexes, lacrimation, and spontaneous movement” [13].

Potassium chloride (2 mEq/kg), in conjunction with general anesthesia, was used to euthanize the animals at the conclusion of the experiment.

“Bilateral femoral (arterial and venous), carotid and jugular approaches were used for multiple sheath insertions using a standard percutaneous Seldinger technique. An initial rapid IV infusion of 1000 mL normal saline was administered after anesthesia induction, followed by a continuous IV drip at a rate of 100 mL/h to 500 mL/h to reach and maintain a mean right atrial pressure of 5 mmHg to 7 mmHg. An unfractionated heparin IV bolus (100 U/kg) was administered after sheath placement, followed by a continuous IV infusion of 50 U/kg/h to maintain an activated clotting time of 200 s to 250 s. Values were monitored every hour using the Hemochron Jr Signature Plus Microcoagulation System (ITC, Piscataway, NJ, USA)” [13][23].

“Ventilation was provided using a ventilator (Hamilton G5, Hamilton Medical AG, Switzerland) set to the INTELLiVENT—Adaptive Support Ventilation mode. The ventilator was set to maintain an oxygen saturation (SpO_2_) of 95% to 99% and an end-tidal carbon dioxide (CO_2_) pressure of 4.8 kPa to 5.6 kPa” [13].

### VA-ECLS

The ECLS circuit consisted of a console (i-cor, Xenios AG, Germany), diagonal blood pump and tubing set, a membrane oxygenator (Xenios AG, Germany), and a mechanical gas blender (Sechrist, USA). A venous cannula (21 Fr, Maquet, Germany) and arterial cannula (18 Fr, Xenios AG, Germany) were inserted percutaneously using the standard Seldinger technique into the femoral vein and artery. The venous inflow cannula was inserted into the right atrium (the tip position was verified using fluoroscopy), and the femoral arterial outflow cannula was inserted into the femoral artery with the tip placed in the descending aorta. Blood gas values leaving the oxygenator were continuously monitored (CDI^™^ Blood Parameter Monitoring System 500, Terumo Cardiovascular Systems Corporation, Elkton, MD, USA). The oxygen/air flow was repeatedly adjusted to maintain a partial pressure of oxygen (*Pa*O_2_) and partial pressure of CO_2_ (*Pa*CO_2_) in the ranges of 10 kPa to 15 kPa, and 4.0 kPa to 6.5 kPa, respectively. The EBF rate was set to 1 L/min until the start of the experiment.

The i-cor system can transition between pulsatile and continuous flow while automatically maintaining the total EBF rate. Pulsatile flow is generated by increasing pump rotation speed during diastole and decreasing the speed during systole. Accordingly, diastolic blood flow increases whereas presystolic and systolic EBF decreases. The changes in pump rotation speed are triggered from the ECG. The pulsatile mode operates in a 1:1 ratio with the heart cycle at a heart rate (HR) of up to 110 beats/min. In the present study, an IV bolus of verapamil (5 mg) was administered, if necessary, to decrease HR to <110 beats/min so that pulsatile flow in a 1:1 ratio with cardiac contractions could be generated.

### Vital function and hemodynamic monitoring

Arterial pressure was measured using standard invasive methods with fluid-filled pressure transducers (Truwave, Edwards Lifesciences LLC, USA) through a pigtail catheter inserted into the aortic arch. A Swan-Ganz catheter was inserted via the femoral vein into the pulmonary artery. Electrocardiographic parameters, HR, invasive blood pressures (aortic arch and central vein), pulse oximetry, capnometry, and invasive central venous SpO_2_ were continuously monitored in all animals (Monitor Life Scope TR, Nihon Kohden, Japan; and Vigilance II, Edwards Lifesciences, USA). Four-channel NIRS oximetry (brain, upper [front] limb, body, lower [hind] limb; INVOS, Medtronic, USA) was used to monitor tissue perfusion; the threshold for the detection of hypoperfusion was 40%.

### Pressure-volume analysis

**Fig 1:**
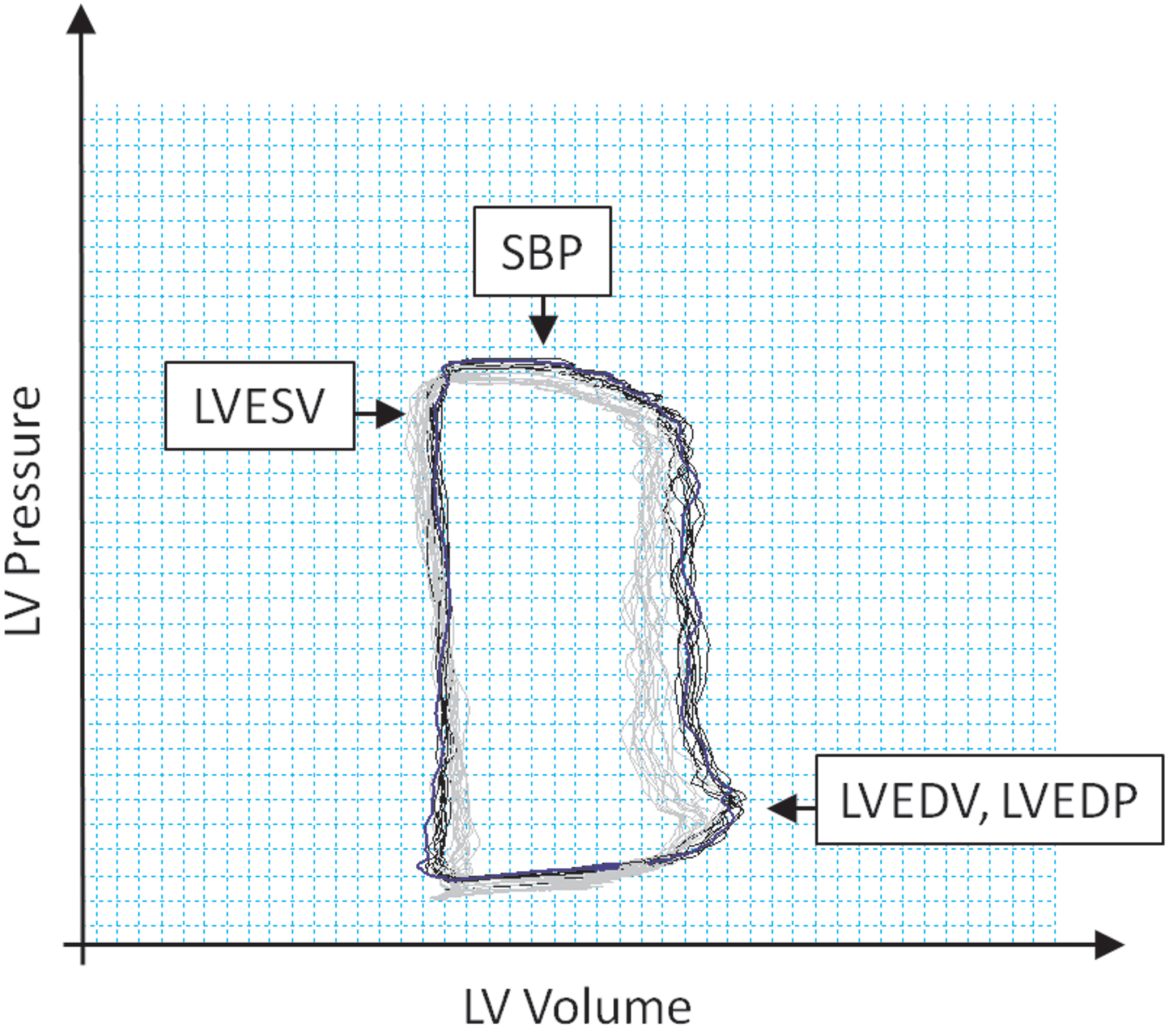
Pressure-volume loop analysis. LV, left ventricle; SBP, systolic blood pressure; LVESV, left ventricular end-systolic volume; LVEDV, left ventricular end-diastolic volume; LVEDP, left ventricular end-diastolic pressure.

A pressure-volume (PV) conductance catheter (Scisense 7 Fr VSL Pigtail, Transonic, USA) was inserted into the left ventricle from the left carotid artery through the aortic valve to monitor cardiac performance during cardiogenic shock induction and maintenance. Correct positioning was assessed radiographically and by verifying optimal PV loop morphology. The catheter was connected to the PV unit (Scisense ADV 500, Transonic, USA) and operated in the admittance mode. The volume was calibrated according to baseline pulmonary thermodilution (Combo CCO catheter, Edwards Lifesciences, USA). PV values were recorded continuously during the experiment. After a brief period for stabilization, the PV values from five end-expiration loops were measured at each EBF level, averaged, and used for analysis. The PV data collected included LV end-diastolic pressure (LVEDP), LV end-diastolic volume (LVEDV), systolic blood pressure (SBP), and LV end-systolic volume (LVESV) (Fig 1).

LV stroke volume (LVSV) was calculated as LVSV = LVEDV − LVESV; LV ejection fraction (LVEF) was calculated as LVEF = LVSV/LVEDV; and cardiac output (CO) was calculated as CO = LVSV × HR.

### Coronary flow measurement

In eight animals, a coronary Doppler guide wire (FloWire, Volcano, USA) was placed in the proximal segment of the left anterior descending coronary artery to measure coronary flow after the development of cardiogenic shock. Average peak flow velocity was recorded continuously during the experiment.

### Cardiogenic shock

After the initiation of ECLS, the animals were stabilized for 10 min. Artificial ventilation was subsequently switched to the CMV mode (5 breaths/min, 100 mL inspiratory volume, and fraction of inspired oxygen 0.21), which precipitated severe hypoxemia in the blood entering the left chambers of the heart and, in turn, caused tissue hypoxia in all tissues perfused by LV ejections, including the coronary arteries. The resulting global myocardial hypoxia rapidly lowered cardiac contractility, LVEF and arterial blood pressure. Concurrently, the lower body was perfused with fully oxygenated blood from the ECLS entering the circulation via the femoral artery. During the hypoxic period, EBF was gradually increased to maintain a mean arterial pressure (MAP) >60 mmHg, thereby ensuring adequate perfusion pressure. After approximately 1 h of myocardial hypoxia, the hemodynamic criteria for severe cardiogenic shock were met, including LVEF, which decreased to <30% and CO, which decreased to <3.5 L/min. Thereafter, continuous perfusion of the heart with hypoxemic blood maintained advanced myocardial dysfunction and severely compromised hemodynamic function. If cardiac performance decreased further with a risk for circulation collapse, the target level of cardiogenic shock severity was adjusted with a partial increase in ventilation [23].

### Experimental protocol

After placement of all catheters and the establishment of ECLS, the animals were allowed to stabilize for 10 min. Cardiogenic shock was induced using global myocardial hypoxia, as described above. Following the occurrence of signs of tissue hypoperfusion (NIRS oximetry <40%) and an additional 10 min of stabilization, the EBF rate was set to 4 L/min and gradually decreased by 0.5 L/min every 10 min. The study had the crossover design, the animals were randomly assigned (by an individual blinded to the study protocol) to start at each EBF rate in continuous mode for 5 min, followed by pulsatile mode for 5 min, or vice versa. Once an EBF rate of 1 L/min was achieved, it was maintained for 5 min. Subsequently, the EBF rate was gradually increased by 0.5 L/min every 10 min, and the mode (continuous to pulsatile, or vice versa) was switched in the middle of this period. At an EBF rate of 4 L/min, the animals were stabilized again for 10 min, and a second cycle of stepwise EBF decrease and increase was performed, and the order of flow mode assessment was reversed. Measurements were performed at the end of each 5 min interval (four data sets per animal), and a mean value was calculated and used in further analysis.

### Statistical analysis

The results are expressed as mean ± SEM. Gaussian (i.e., normal) distribution of all data sets was tested using the D’Agostino-Pearson normality test. The primary experimental variables in the present study were EBF and the mode of EBF (i.e., pulsatile or continuous). The study was fully factorial (i.e., each animal was tested in all possible conditions); accordingly, the data were analyzed using two-way repeated measured ANOVA. When ANOVA testing indicated significant differences among EBFs and/or flow modes, specific pre-planned comparisons were performed, and P-values were adjusted according to the Bonferroni method to maintain the experiment-wise α level at ≤ 0.05; P<0.05 was considered to be statistically significant. All statistical analyses were performed using GraphPad Prism version 5.0 (GraphPad, San Diego, CA, USA).

## Results

All animals survived and completed all protocol-defined procedures. Myocardial hypoxia resulted in a decline in CO, SBP and LVEF (Table 1). Two animals required the administration of verapamil to decrease HR to <110 beats/min.

**Table 1:** Major hemodynamic variables at baseline and after the development of cardiogenic shock.

	CO (L/min)	SBP (mmHg)	MAP (mmHg)	HR (beats/min)	LVEF (%)
Baseline	6.6±1.1	115.7±8.2	90.5 ±3.9	78.4 ±4.0	61.2 ±4.3
Cardiogenic shock	1.7±0.7	63.5 ±22.2	33.8±10.9	105.5±2.5	21.9±6.7

CO, cardiac output; HR, heart rate; MAP, mean arterial pressure; LVEF, left ventricular ejection fraction; SBP, systolic blood pressure

**Fig 2:**
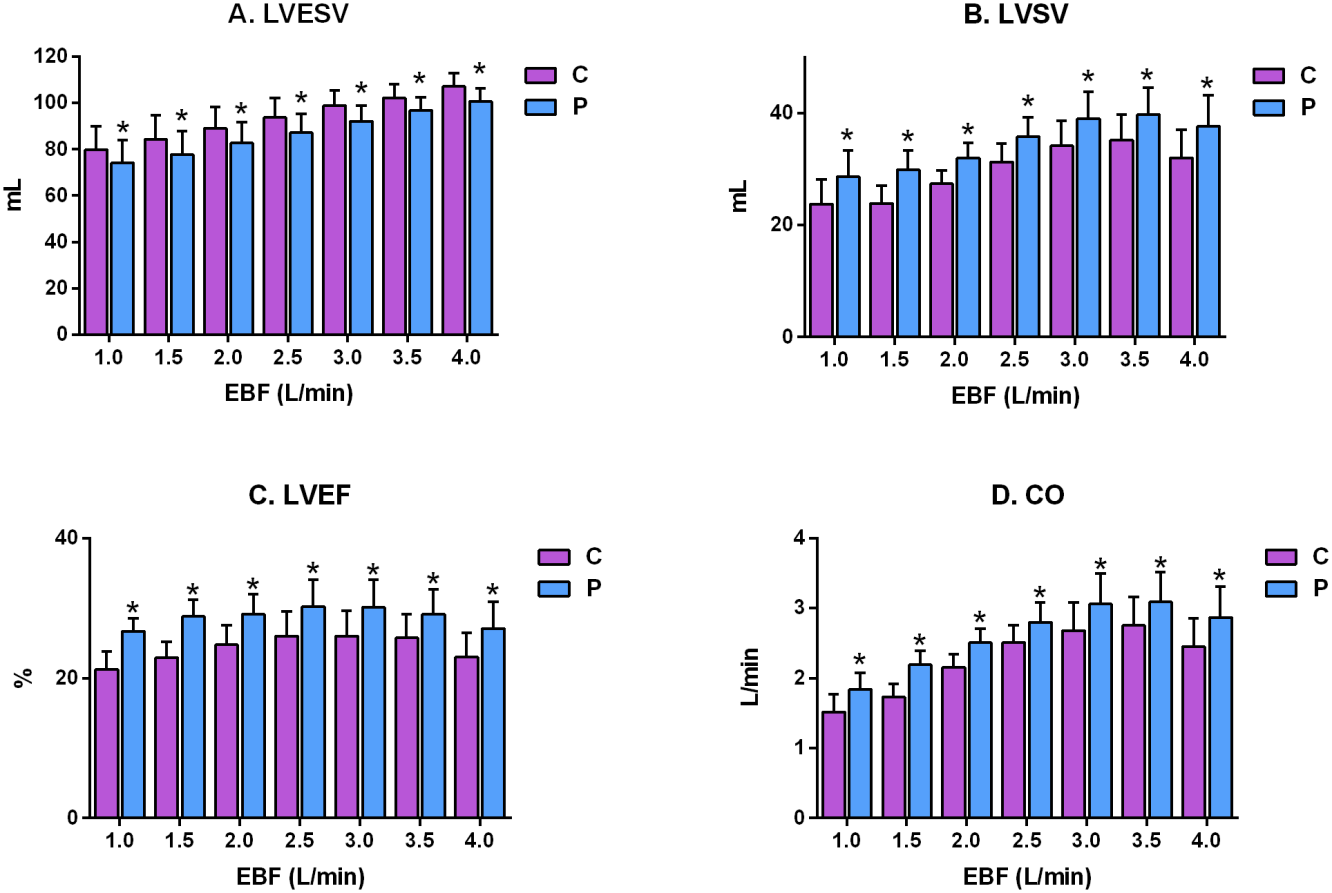
Comparison of the effect of different levels of continuous (C) and synchronized pulsatile (P) extracorporeal blood flow (EBF) on left ventricular performance parameters in a porcine model of cardiogenic shock. (A) LVESV, left ventricle end-systolic volume; (B) LVSV, left ventricle stroke volume; (C) LVEF, left ventricle ejection fraction; (D) CO, cardiac output. *P<0.05.

Synchronized pulsatile flow was present at all EBF rates, apparent as an increase in the diastolic pressure wave form immediately after the diacrotic notch, and was associated with a significant reduction in LVESV, on average, by 6.25 mL (6.7%; P < 0.0001 compared with continuous EBF across all flow rates). There were also significant differences between pulsatile and continuous EBF (5.6±0.8, 6.6±1.0, 6.2±0.9, 6.6±0.5, 6.7±0.9, 5.5±0.7 and 6.4±0.7 mL) at EBF rates of 1.0, 1.5, 2.0, 2.5, 3.0, 3.5 and 4.0 L/min, respectively, as shown in Fig 2A. On average, LVSV significantly increased by 5.0 mL (17.4%; P<0.0001 compared with continuous EBF across all flow rates). At each particular flow rate, LVSV also increased significantly (4.9±0.8, 6.0±1.0, 4.6±0.9, 4.5±0.7, 4.9±0.9, 4.6±0.5 and 5.6±0.8 mL) at each increment of the respective EBF rates (Fig 2B). On average, LVEF significantly increased during pulsatile flow by 4.5% (18.8%; P < 0.0001 compared with continuous EBF across all flow rates). At each particular flow rate, LVEF also increased significantly (5.4±0.8%, 5.9±0.7%, 4.3±0.7%, 4.2±0.5%, 4.1±0.6%, 3.4±0.3%, and 4.1±0.5%) at each incremental increase in EBF (Fig 2C). Finally, CO was significantly increased, on average, by 0.37 L/min (17.1% P < 0.0001 compared with continuous EBF across all flow rates), and CO was also increased significantly at each of the individual EBFs, respectively (0.32±0.05, 0.46±0.05, 0.36±0.08, 0.28±0.1, 0.38±0.07, 0.34±0.06 and 0.42±0.06 L/min), as shown in Fig 2D.

**Fig 3:**
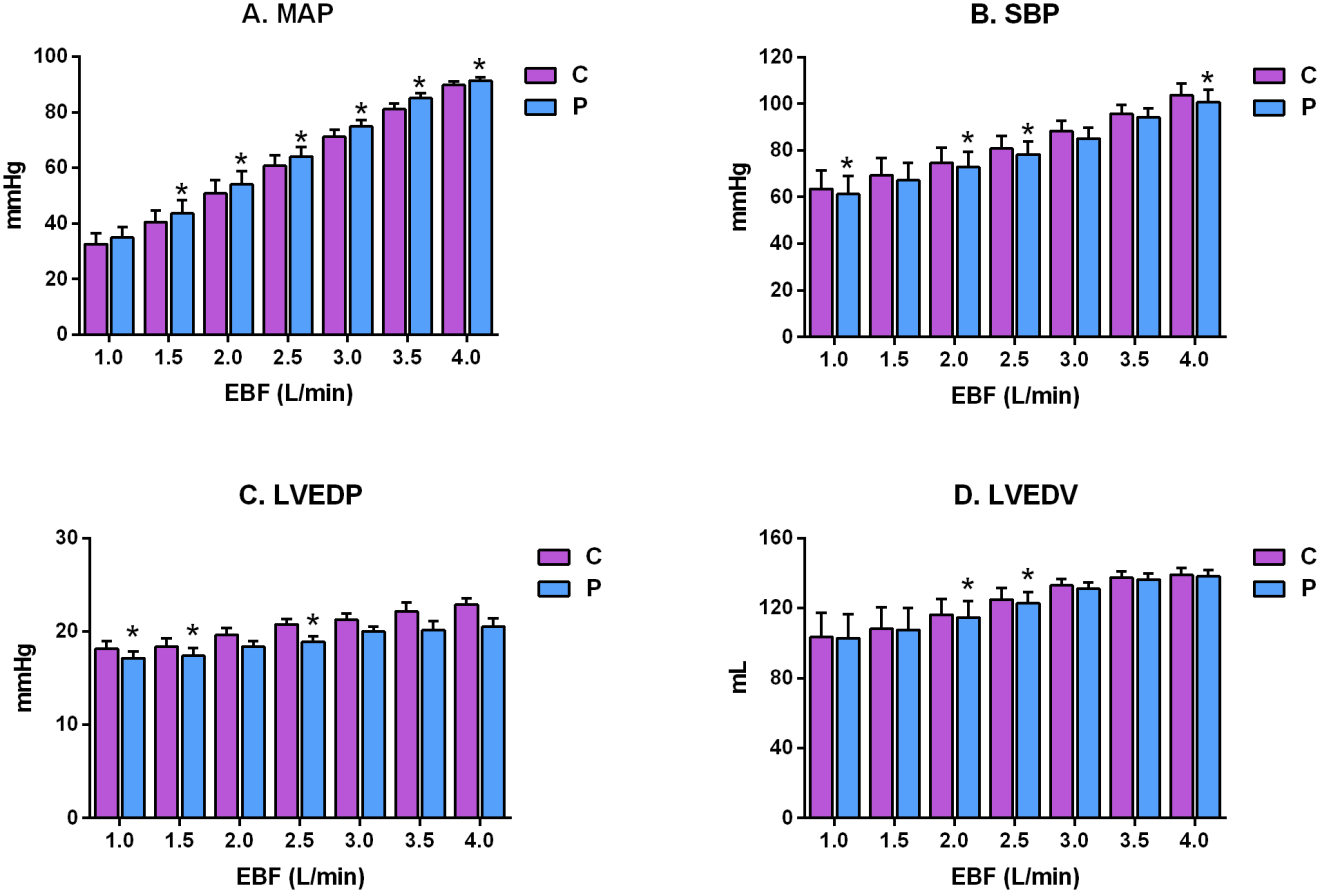
Comparison of the effect of different levels of continuous (C) and synchronized pulsatile (P) extracorporeal blood flow (EBF) on hemodynamic and left ventricular performance parameters in a porcine model of cardiogenic shock. (A) MAP, mean arterial pressure; (B) SBP, systolic blood pressure; (C) LVEDP, left ventricle end-diastolic pressure; (D) LVEDV, left ventricle end-diastolic volume. *P<0.05.

In terms of cardiovascular pressures, pulsatile EBF significantly increased MAP by an average of 3.0 mmHg (5.5%) across all flow rates compared with continuous EBF (P<0.001). Moreover, MAP rose significantly as a linear function of EBF during both continuous and pulsatile flow (P<0.001). However, the increase in MAP at each flow rate was variable: MAP increased significantly at all individual EBF values (2.2±0.6, 3.1±1.0, 3.2±0.7, 3.4±0.7, 3.8±1.1, 4.0±1.2 and 1.5±0.6 mmHg) except at an EBF of 4.0 L/min, at which the increase in measured MAP was not statistically significant (Fig 3A). Similarly, the SBP was significantly lower during the pulsatile flow mode, on average, compared with continuous EBF (P<0.0001), and SBP rose significantly as EBF increased during both continuous and pulsatile flow conditions (P = 0.0004). When compared at each EBF, the reduction in SBP during the pulsatile mode compared with continuous mode was significant (P<0.05) only at EBF rates of 1.0, 2.5, 3.0 and 4.0 L/min, as shown in Fig 3B. There was a significant reduction in LVEDP, on average, across all EBF levels (P<0.001), and LVEDP rose monotonically as EBF increased from 1.0 L/min to 4.0 L/min (P = 0.0021). When comparisons were performed at each EBF, there was a significant reduction in LVEDP during pulsatile compared with continuous EBF at 2.5, 3.5 and 4.0 L/min (P<0.05) (Fig 3C). Finally, LVEDV was significantly decreased across all EBF rates during pulsatile compared with continuous EBF, and the LVEDV increased as the EBF increased during both pulsatile and continuous modes (P = 0.0401). Nevertheless, the decrease in LVEDV that we observed during the pulsatile mode compared with the continuous mode was only significant at EBF values of 3 L/min and 2.5 L/min (P<0.05) (Fig 3D). HR was not influenced by the mode of ECLS flow.

**Fig 4:**
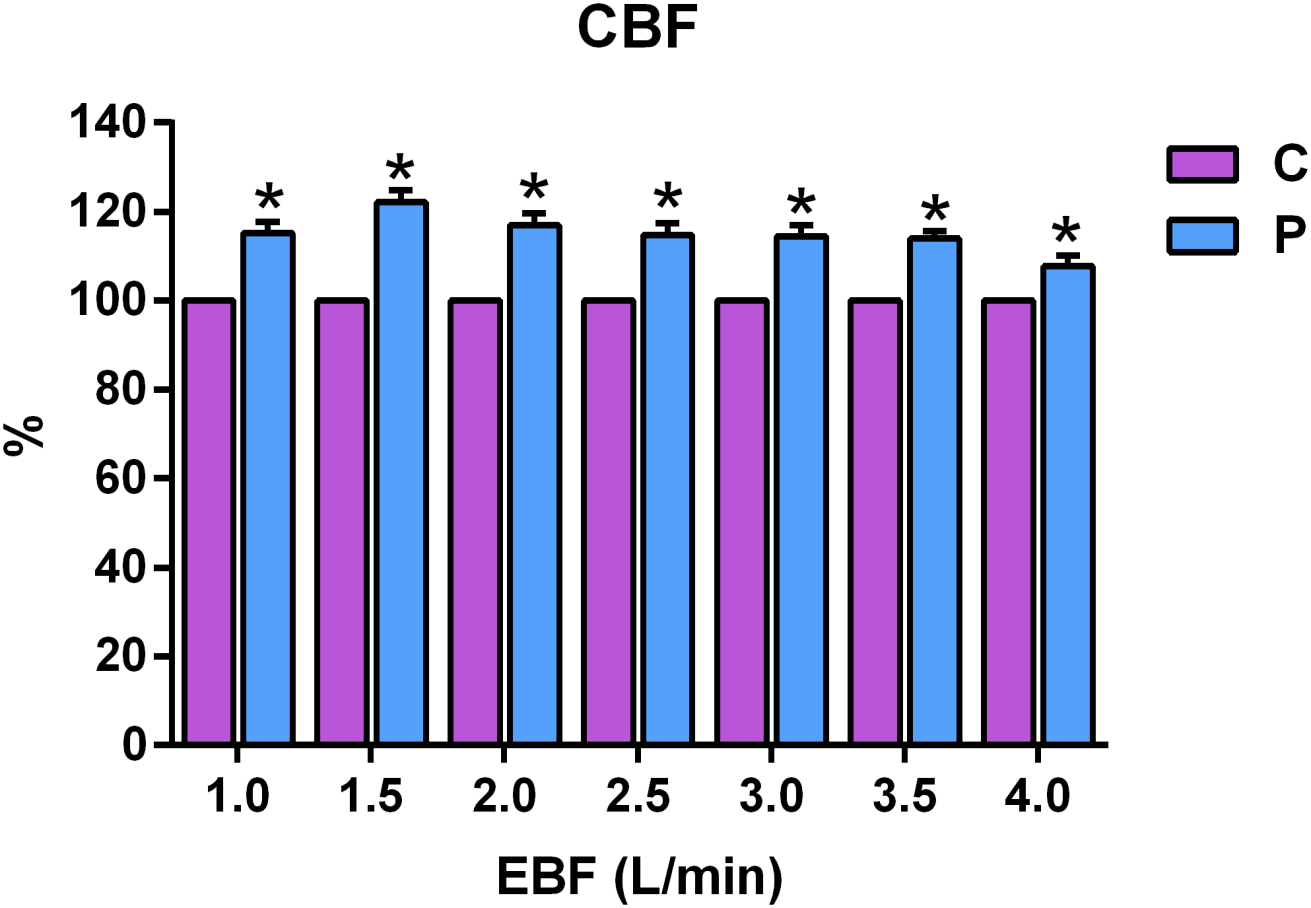
Comparison of the effect of different levels of continuous (C) and electrocardiogram-synchronized pulsatile (P) extracorporeal blood flow (EBF) on coronary blood flow measured using an intracoronary Doppler wire in a porcine model of cardiogenic shock. *P<0.05.

Compared with continuous ECLS flow, ECG-synchronized pulsatile flow was associated with significantly increased coronary flow velocity across all EBF values (P<0.001), and when compared at each flow rate, coronary flow velocity increased by 15.2±2.6%, 22.1±2.7%, 17.0±2.7%, 14.8±2.7%, 14.6±2.4%, 13.9±1.6% and 7.8±2.4%, respectively, at EBF rates of 1.0, 1.5, 2.0, 2.5, 3.0, 3.5 and 4.0 L/min (P<0.05) (Fig 4).

## Discussion

To the best of our knowledge, this was the first study to focus on a direct comparison of synchronized pulsatile and standard continuous flow ECLS using an animal model of acute cardiogenic shock. The major finding of the present study was the observation of significant increases in LVSV, LVEF, CO and MAP, and marked reduction in LVESV during pulsatile ECLS flow compared with continuous flow at each respective EBF rate. These results suggest that ECG-synchronized, pulsatile EBF, with increased diastolic and decreased systolic flow, may improve LV unloading and decrease the risk for LV overload in cases of severe cardiogenic shock treated with ECLS.

VA-ECLS is a minimally invasive circulatory support system that enables the maintenance of adequate tissue perfusion, even in situations of critical circulatory collapse such as cardiac arrest. It has been shown that the hemodynamic efficacy of VA-ECLS is superior to other percutaneous circulatory support devices such as the Impella 2.5 (Abiomed, Danvers, MA, USA) or TandemHeart (Cardiac Assist Inc, Pittsburg, PA, USA) [24]. However, significant increases in arterial blood pressure caused by VA-ECLS are also associated with an elevation in LV afterload. In the presence of severe systolic dysfunction, increased LV afterload may have a detrimental effect on LV function(s), resulting in LV overload with LV distension, increased wall stress, reduction of stroke volume, increased left atrial pressure and, eventually, the development of severe pulmonary edema. The impairment of LV performance with increasing EBF has been documented in several animal [13] and human [20, 25] studies. Therefore, VA-ECLS primarily represents a circulatory support system rather than an LV-assist device.

Several approaches have been proposed to treat LV overload under VA-ECLS therapy for cardiogenic shock. The most efficient solution to this scenario appears to be the transition from mechanical VA-ECLS circulatory support to an LV-assist device (possibly including an oxygenator in the circuit), with the insertion of the inflow cannula into the LV apex and outflow cannula to the aorta. This approach, however, is often not a suitable option, for example, in cases of acute large myocardial infarction affecting the LV apex; alternative inflow cannulation of the left atrium may not necessarily unload the left ventricle. Several minimally invasive methods have also been demonstrated to unload the left ventricle under VA-ECLS therapy including atrial septostomy, left atrial unloading, or insertion of an intra-aortic balloon pump or Impella device [26]. These interventions are, however, also associated with additional invasive access, method-related complications, and/or higher costs. In contrast, the pulsatile ECLS system may offer full ECLS circulatory support with decreased LV afterload and improved LV unloading, without the need for any additional vascular puncture or additional costs. Poss et al [27] described the first-in-man successful use of the i-cor system in a group of patients with severe refractory cardiogenic shock.

Our results are consistent with a previous study that used the i-cor pulsatile ECLS system to investigate increased coronary artery flow by comparing pulsatile flow with continuous EBF [28]. However, in contrast with the study by Cremers et al [28], we observed increased MAP. This difference could be explained by the difference in models used: Cremers et al [28] focused on ventricular fibrillation (with a fixed schedule of pulsatility unrelated to the ECG), whereas we studied the effects of the i-cor system in a cardiogenic shock model, in which the pulsatile flow was synchronized with the heartbeat, and each of the increments in pulsatile flow were also associated with increased CO compared with the continuous flow mode.

Several authors have suggested that the pulsatile mode may also be associated with improved systemic microcirculation perfusion and superior end-organ protection [21, 29, 30]. Because our study was focused entirely on the heart, we cannot directly support this observation; however, we speculate that the higher MAP observed in our study may have been associated with improved end-organ perfusion. We are also unable to confirm the findings from published in vitro studies using the i-COR system, which demonstrated improved transmission of hemodynamic energy with pulsatile flow [22, 31].

The present study had several limitations, the first of which were those inherent to the i-cor device. Although the changes in cardiac function are reasonably consistent across all flow rates, there is a tendency for the optimum values of cardiac function to occur in the EBF range from 2.5 L/min to 3.5 L/min (see LVSV, LVEF and CO in Fig 2). At 4.0 L/min, the benefit of pulsatile flow compared with continuous EBF tended to diminish (see MAP and LVEDV in Fig 3). The diagonal pump in the i-cor device has a maximum rotational speed of 8000 rpm. Some of the available rotational energy is used to generate the baseline flow rate and, as the baseline EBF increases, the rotational reserve diminishes, and there is less diagonal pump reserve (the increment of pulsatile increase in rotational speed diminishes as more of the rotational energy is used to generate the baseline flow rate). The distribution of pump function between the continuous baseline value and the pulsatile addition during diastole, therefore, appears to be optimal between 2.5 L/min and 3.5 L/min.

The animal model may also present certain limitations. We used a global hypoxia model of cardiogenic shock, affecting not only the left but also the right ventricle, and causing severe dysfunction in both ventricles. In this respect, our model reflects frequent clinical scenarios and significantly differs from other large animal models of acute severe heart failure, which are primarily based on coronary artery occlusions. This difference may have also contributed to the non-linear impairment of LV performance variables with increasing EBF rates in the present study. In our previous study using a porcine model of cardiogenic shock induced by the perfusion of the coronary artery with deoxygenated venous blood, LV function gradually diminished with increasing EBF rates [13]. This discordance could, however, also be explained by the extremely low perfusion pressures at the lowest EBF rates in the present study. Furthermore, LV ejections in the model described herein not only supply the coronary arteries with hypoxemic blood but, at least during the initial phases of the hypoxic period, also other tissues including the brain; therefore, cerebral damage could be anticipated. We cannot exclude the possibility that central nervous system hypoxia may have impaired some of the reflexes regulating blood circulation. Moreover, verapamil was used in two animals to decrease HR to <110 beats/min to allow pulsatility of the i-cor system in a 1:1 ratio. Although verapamil can also influence cardiac contractility, it is unlikely that it was the cause of the differences between pulsatile and continuous EBF. Another limitation originates from the high sensitivity of the coronary Doppler wire to even small changes in position in the coronary artery. Accordingly, the wire’s position had to be repeatedly adjusted before measurement of coronary flow to obtain a good-quality signal. Depending on the coronary anatomy and wire position, we observed marked inter-individual variability in average peak flow velocity values. However, during measurements, wire positioning remained unchanged and, therefore, intra-individual differences between pulsatile and continuous mode values were likely not affected; accordingly, the results are presented in relative terms.

Only selected hemodynamic and cardiac performance variables were analyzed in our study, which is another significant limitation. We realize that other parameters such as dP/d*t*_max_ or NIRS oximetry values could provide additional information, however, these data were not recorded or calculated.

## Conclusion

Our results suggest that ECG-synchronized pulsatile ECLS flow preserves LV function (and may actually unload the LV) and increases coronary flow, CO, and MAP compared with standard continuous-flow ECLS in a porcine model of severe acute cardiogenic shock. These beneficial effects of ECG-synchronized pulsatile ECLS could be reflected in the improvement of circulatory conditions in cases of severe or rapidly progressing cardiogenic shock with markedly impaired left ventricular function treated with ECLS.

## Supporting information

S1 File Data set. This is the full data set used in this study.
(XLSX)
